# Supplementary material for: Coagulation and Fibrinolysis in Obstructive Sleep Apnoea
Source: Int J Mol Sci. 2021 Mar 11;22(6):2834. doi: 10.3390/ijms22062834 (PMC8000922; doi:10.3390/ijms22062834)
Supplement: Supplementary file 1 [file ijms-22-02834-s001.pdf]

International Journal of *Molecular Sciences*

MDPI

Supplementary materials

Table 1. OSA and its therapy on coagulation cascade and its regulators.

Factor	Ref.	Study design	Population	Therapy	Main findings
**Coagulation cascade**					
**TF**	[83]	Case-control / Interventional	n=35 with OSA<br>n=12 control<br>(only males)	8 weeks of CPAP	- TF higher in OSA group compared to the controls<br>- No correlation between vWF levels and AHI, AI<br>- TF positively associated with TST90%<br>TF decreased after CPAP
	[84]	Case control	n=60 with OSA *(AHI≥10/h)*<br>n=30 control<br>(only males)	n/a	- TF higher in OSA group compared to the controls<br>- TF higher in the non-obese OSA subgroup compared to the controls<br>- TF increased with severity<br>- TF correlated with ODI
	[85]	Case control	n=19 with OSA *(AHI≥15/h)*<br>n=116 control	n/a	- TF associated with less sleep efficiency and wake after sleep onset<br>- TF was predicted by more disrupted sleep and the indexes of poor sleep efficiency
**VII**	[86]	Randomised controlled	n=220 with OSA *(ODI>4%>10)*<br>n=112 subtherapeutic CPAP<br>n=108 therapeutic CPAP<br>n=32 control	1 month of CPAP	- VIIa higher in OSA group compared to the controls<br>- No correlation with severity<br>- No change in VIIa levels after CPAP
	[155]	Randomized-controlled	n=28 with OSA	2 months of CPAP	- No change in VIIa levels after CPAP
	[156]	Interventional	n=15 with OSA	6 months of CPAP	- VIIa decreased after CPAP

**XII**	[86]	Randomised controlled	n=220 with OSA *(ODI>4%>10)*<br>n=112 subtherapeutic CPAP<br>n=108 therapeutic CPAP<br>n=21 control	1 month of CPAP	- XIIa higher in OSA group compared to the controls<br>- No correlation with severity<br>- No change in XIIa levels after CPAP
**VIII**	[86]	Randomised controlled	n=220 with OSA *(ODI>4%>10)*<br>n=112 subtherapeutic CPAP<br>n=108 therapeutic CPAP	1 month of CPAP	- No change in VIII levels after CPAP
**Fibrinogen**	[86]	Randomised controlled	n=220 with OSA *(ODI>4%>10)*<br>n=112 subtherapeutic CPAP<br>n=108 therapeutic CPAP	1 month of CPAP	- No change in fibrinogen levels after CPAP
	[87]	Cross-sectional	n=144 with OSA (AHI≥15/h)<br>n=392 control (from Cleveland Family Study cohort)	n/a	- Increase in fibrinogen associated with increase in AHI<br>- Fibrinogen increased by 8.4 mg/dl per 5-unit AHI increase until an AHI of 15
	[88]	Cross-sectional	n=79 with OSA *(AHI≥5/h)*<br>n=1079 control	n/a	- Fibrinogen higher in OSA group compared to the controls
	[89]		n=113 with ischemic stroke<br>n=69 with OSA *(RDI≥5/h)*<br>n=44 control	n/a	- Fibrinogen higher in OSA group compared to the controls<br>- Fibrinogen correlated with RDI and longest apnoea duration

					- Fibrinogen negatively correlated with ODI, average $MinSatO_2$, $MinSatO_2$<br>- Average $MinSatO_2$ and gender independent predictor for fibrinogen levels
	[90]	Case control	n=52 with OSA *(AHI≥5/h)*<br>n=30 control	n/a	- Fibrinogen did not differ between the two groups<br>- No correlation between fibrinogen and AHI
	[140]	Interventional	n=13 with OSA on CPAP<br>n=8 control	1 night without CPAP	- No change after CPAP
	[155]	Randomized-controlled	n=28 with OSA	2 months of CPAP	- No change after CPAP
	[157]	Interventional	n=32 with OSA	8 weeks of CPAP	- No difference between fibrinogen levels between ≥4h/night and <4h/night CPAP usage
	[158]	Interventional	n=11 with OSA *(AHI≥20/h)*	1 night of CPAP	- Fibrinogen decreased after CPAP
	[168]	Interventional	n=61 with OSA *(AHI≥5/h)*	Upper airway surgery	- Fibrinogen decreased after upper airway surgery
**Regulators**					
**APC**	[93]	Case control	n=269 with OSA *(AHI≥5/h)*<br>n=24 control	n/a	- APC did not differ between the two groups<br>- No correlation with AHI and ODI
**TAT**	[86]	Case control	n=220 with OSA (ODI>4%>10)<br>n=112 subtherapeutic CPAP<br>n=108 therapeutic CPAP<br>n=29 control	n/a	- TAT higher in OSA group compared to the controls<br>- No correlation with severity<br>- No change in TAT levels after CPAP
	[93]	Case control	n=269 with OSA *(AHI≥5/h)*<br>n=24 control	n/a	- TAT higher in OSA group compared to the controls<br>- No correlation with AHI and ODI

	[114]	Case control	n=64 with PE and OSA<br>n=41 with OSA without PE	n/a	- TAT did not differ between the two groups<br>- TAT correlated with ESS
**EPCR**	[111]	Case control	n=198 with OSA *(AHI≥5/h)*<br>n=42 control for blood<br><br>n=197 with OSA *(AHI≥5/h)*<br>n=46 control for urine	n/a	- Blood and urinary EPCR was higher in OSA group compared to the controls
**Thrombomodulin**	[93]	Case control	n=269 with OSA *(AHI≥5/h)*<br>n=24 control	n/a	- Thrombomodulin did not differ between the two groups (only a trend to be higher in OSA)
**Apolipoprotein H**	[113]	Case control	n=50 OSA *(AHI≥5/h)*<br>n=30 control	n/a	- ApoH higher in OSA group compared to the controls<br>- ApoH positively correlated with AHI, $TSat_{90}$ and negatively correlated with mean $SatO_2$
**Annexin V**	[116]	Case control / Interventional	n=14 with OSA *(AHI≥5/h)*<br>n=10 control	8 weeks of CPAP	- Annexin V+ endothelial cells were higher in OSA group compared to the controls<br>- Correlation with AHI<br>- The number of Annexin V+ endothelial cells reduced after CPAP
	[117]	Case control / Interventional	n=18 with OSA *(AHI≥5/h)*<br>n=9 control	2 months of CPAP	- Annexin V+ microparticles were higher in OSA group compared to the controls<br>- Reduced diurnal variability after CPAP
	[118]	Case control / Interventional	n=82 OSA *(AHI≥5/h)*<br>n=22 control	4-6 weeks of CPAP	- Annexin V were higher in OSA group compared to the controls<br>- Annexin V was correlated with carotid intima-media thickness

	- Annexin V was predicted by OSA severity<br>- No changes after CPAP

Table 2. OSA and its therapy on fibrinolysis and its regulators.

Factor	Ref.	Study design	Population	Therapy	Main findings
**Plasminogen activators**					
**tPA**	[96]	Case control	n=13 with OSA<br>n=10 control	n/a	- tPA activity did not differ between the two groups
	[95]	Case control	n=17 with OSA *(AHI≥5/h)*<br>n=17 control	n/a	- tPA did not differ between the two groups<br>- tPA correlated with AI<br>- tPA had a circadian activity
	[97]	Case control / Interventional	n=39 with OSA *(AHI≥5/h)*<br>n=14 control	1 month of CPAP	- tPA was higher in OSA group compared to the controls<br>- No change after CPAP
**uPA**	[97]	Case control / Interventional	n=39 with OSA *(AHI≥5/h)*<br>n=14 control	1 month of CPAP	- uPA was lower in OSA group compared to the controls<br>- No change after CPAP
**suPAR**	[104]	3 years follow-up	n=329 (no sleep study)	n/a	- suPAR was relative to persistently low OSA risk
	[105]	Case control	n=53 with OSA<br>n=15 control	n/a	- sUPAR did not differ between the two groups<br>- No correlation with AHI<br>- sUPAR was higher in women
**XII**	[86]	Randomised controlled	n=220 with OSA *(ODI>4%>10)*<br>n=108 therapeutic CPAP<br>n=112 subtherapeutic CPAP<br>n=26 control	1 month CPAP	- XIIa was higher in OSA group compared to the controls<br>- No correlation with AHI<br>- XII decreased after CPAP
**Kallikrein**	[103]	Case control	n=60 with OSA *(AHI≥1/h)*<br>n=30 with snoring<br>n=31 control<br>(only children)	n/a	- Kallikrein was lower in OSA group compared to the controls
**Inhibitors of fibrinolysis**					
**PAI-1**	[96]	Case control	n=13 with OSA<br>n=10 control	n/a	- PAI-1 was higher in OSA group compared to the controls
	[126]	Case control / Interventional	n=22 with OSA *(AHI≥5/h)*<br>n=16 control	3 months and 6 months after mandibular advancement splint	- PAI-1 did not differ between the two groups<br>- PAI-1 decreased after mandibular advancement splint

[155]	Randomized-controlled	n=28 with OSA	2 months CPAP	- No change after CPAP
[159]	Double-bind randomized	n=44 with OSA (AHI≥15/h)	2 weeks of CPAP	- PAI-1 correlated with AHI<br>- PAI-1 was predicted by AHI<br>- PAI-1 decreased after CPAP
[121]	Randomized-controlled	n=51 with OSA *(AHI≥10/h)*<br>n=24 control	3 weeks of CPAP	- PAI-1 was higher in OSA group compared to the controls<br>- No change after CPAP
[95]	Case control	n=17 with OSA *(AHI≥5/h)*<br>n=17 control	n/a	- PAI-1 was higher in OSA group compared to the controls<br>- PAI-1 activity correlated with AHI, minimum oxygen levels during sleep, minimum oxygen levels<br>- PAI-1 had a circadian activity
[97]	Case control / Interventional	n=39 with OSA *(AHI≥5/h)*<br>n=14 control	1 month of CPAP	- PAI-1 was higher in OSA group compared to the controls<br>- PAI-1 correlated with AHI, ODI, TST90%<br>- PAI-1 decreased after CPAP
[124]	Case control	n=38 with OSA *(AHI≥10/h)*<br>n=22 control	n/a	- PAI-1 was higher in OSA group compared to the controls
[122]	Case control	n=24 with OSA *(AHI≥5/h)*<br>n=29 control	n/a	- PAI-1 activity and antigen was higher in OSA group compared to the controls<br>- PAI-1 activity correlated with AHI
[123]	Case control	n=21 with OSA *(AHI≥15/h)*<br>n=22 control	n/a	- PAI-1 was higher in OSA group compared to the controls
[119]	Case control	n=45 with OSA *(AHI≥5/h)*<br>n=19 control	n/a	- PAI-1 was higher in OSA group compared to the controls<br>- PAI-1 levels increased with the severity<br>- PAI-1 positively correlated with AHI, AI, RDT, DI, BMI and negatively correlated with meanSpO_2 and minSpO_2
[120]	Case control	n=9 with OSA *(AHI≥5/h)*<br>n=9 control	n/a	- PAI-1 tended to be higher in OSA group compared to the controls

	[125]	Case control	n=75 with OSA *(RDI≥3/h)* n=129 control (only children)	n/a	- PAI-1 was higher in OSA group compared to the controls
	[127]	Case control	n=55 OSA *(AHI≥5/h)* n=125 control	n/a	- PAI-1 was higher in OSA group compared to the controls<br>- PAI-1 correlated with AHI<br>- PAI-1 was predicted by the interaction between AHI and metabolic syndrome
	[160]	Interventional	n=124 with OSA *(AHI≥1/h)*	6 months after adenotonsillectomy	- PAI-1 decreased after adenotonsillectomy
	[161]	Interventional	n=20 with morbid obesity n=14 with OSA	1 year after sleeve gastrectomy	- PAI-1 decreased after sleeve gastrectomy
**Klotho**	[131]	Case control	n=21 with OSA *(AHI≥5/h)* n=41 control	n/a	- Klotho was lower in OSA group compared to the controls<br>- No correlation with AHI, TST, AI<br>- Klotho correlated with ODI, TST90%, minSatO_2
**A2AP**	[119]	Case control	n=45 with OSA *(AHI≥5/h)* n=19 control	n/a	- A2AP was higher in OSA group compared to the controls<br>- A2AP levels increased with the severity<br>- A2AP positively correlated with AHI, AI, RDT, DI and negatively correlated with meanSpO_2 and minSpO_2
**TAFI**	[126]	Case control / Interventional	n=22 with OSA *(AHI≥5/h)* n=16 control	3 months and 6 months after mandibular advancement splint	- TAFI was higher in OSA group compared to the controls<br>- TAFI decreased after mandibular advancement splint

Table 3. OSA and its therapy on platelet function.

Factor	Ref.	Study design	Population	Therapy	Main findings

**Platelet activation and aggregation**	[136]	Case control	n=42 with OSA *(AHI≥10/h)*<br>n=23	1 night / 3 months of CPAP	- Platelet activation decreased after 1 night and 3 months CPAP<br>- Platelet activation correlated with AHI, AI, ESS and log $SatO_2$<90%<br>- AI and gender independent predictor for platelet activation
	[137]	Case control / Interventional	n=12 with OSA *(AHI≥5/h)*<br>n=12 control	8 weeks of CPAP	- Platelet aggregation was higher in OSA group compared to the controls<br>- Platelet aggregation correlated with TST90%<br>- Platelet aggregation decreased after CPAP
	[138]	Case control / Interventional	n=6 with OSA *(AHI≥5/h)*<br>n=5 control	1 night of CPAP	- Platelet activation did not differ between the two groups<br>- Platelet activation and aggregation decreased after CPAP
	[139]	Case control / Interventional	n=12 with OSA *(AHI≥10/h)*<br>n=6 control	1 night of CPAP	- Platelet activation was higher in OSA group compared to the controls<br>- No change in platelet activation after CPAP (only a trend to reduced activation)
	[140]	Case control / Interventional	n=13 with OSA on CPAP<br>n=8 control	1 night without CPAP	- Platelet activity did not differ between controls and patients with or without CPAP therapy
	[96]	Case control	n=13 with OSA<br>n=10 control	n/a	- Platelet aggregation did not differ between the two groups
	[163]	Interventional	n=41 with OSA *(AHI≥15/h)*	30 days of CPAP	- Platelet aggregation decreased after CPAP
	[164]	Case control / Interventional	n=58 with OSA *(ODI>3%>15)*<br>n=66 control	90 days of CPAP	- Platelet aggregation was higher in OSA group compared to the controls<br>- Platelet aggregation decreased after CPAP
**GP-Ib**	[62]	Case control	n=47 with OSA *(AHI≥10/h)*<br>n=30 control	n/a	- GP-Ib did not differ between the two groups, but downregulated after ADP stimulation<br>- TST90% and gender were independent predictor for GP-Ib levels

**GP IIb-IIIa**	[62]	Case control	n=47 with OSA *(AHI≥10/h)*<br>n=30 control	n/a	- GP-IIb-IIIa did not differ between the two groups
**vWF**	[83]	Case control / Interventional	n=35 with OSA<br>n=12 control<br>(only males)	8 weeks of CPAP	- vWF higher in OSA group compared to the controls<br>- No correlation between vWF levels and AHI, AI<br>- No change in vWF levels after CPAP
	[85]	Case control	n=19 with OSA *(AHI≥15/h)*<br>n=116 control	n/a	- vWF associated with less sleep efficiency and wake after sleep onset<br>- vWF was associated with sleep fragmentation and disturbances in sleep architecture<br>- vWF was predicted by more disrupted sleep, indexes of sleep fragmentation and disturbed sleep architecture
	[86]	Randomised controlled	n=220 with OSA *(ODI>4%>10)*<br>n=108 therapeutic CPAP<br>n=112 subtherapeutic CPAP<br>n=32 control	1 month of CPAP	- vWF levels did not differ between OSA and control groups<br>- No correlation with severity<br>- No change in vWF levels after CPAP
	[104]	3 years follow-up	n=329 (no sleep study)	n/a	- Increase in vWF was associated with new-onset OSA risk
	[145]	Case control	n=41 with OSA<br>n=41 control	n/a	- vWF did not differ between the two groups<br>- No correlation with oxygen desaturation
	[155]	Randomized-controlled	n=28 with OSA	2 months of CPAP	vWF decreased after CPAP
	[159]	Randomized-controlled	n=44 with OSA *(AHI≥15/h)*	2 weeks of CPAP	- No change after CPAP
	[121]	Randomized-controlled	n=51 with OSA *(AHI≥10/h)*<br>n=24 control	3 weeks of CPAP	- No change after CPAP
	[165]	Interventional	n=44 with OSA *(AHI≥15/h)*	1.72 years after upper airway surgery	- vWF decreased after upper airway surgery

**P-selectin**	[62]	Case control	n=47 with OSA *(AHI≥10/h)*<br>n=30 control	n/a	- P-selectin did not differ between the two groups<br>- No correlation with the markers of OSA
	[86]	Randomised controlled	n=220 with OSA *(ODI>4%>10)*<br>n=108 therapeutic CPAP<br>n=112 subtherapeutic CPAP<br>n=32 control	1 month of CPAP	- P-selectin was higher in OSA group compared to the controls<br>- Correlation with BMI<br>- No change after CPAP
	[117]	Case control	n=18 with OSA *(AHI≥5/h)*<br>n=9 control	n/a	- P-selectin did not differed between OSA and control groups
	[126]	Case control / Interventional	n=22 with OSA *(AHI≥5/h)*<br>n=16 control	3 months and 6 months after mandibular advancement splint	- P-selectin was higher in OSA group compared to the controls<br>- No changes after mandibular advancement splint
	[142]	Case control	n=51 with OSA *(AHI≥5/h)*<br>n=42 control	n/a	- P-selectin did not differed between OSA and control groups<br>- P-selectin was higher in severe OSA group compared to the mild OSA group and the control group
	[143]	Case control	n=48 with OSA *(AHI≥5/h)*<br>n=16 control	n/a	- P-selectin was higher in OSA group compared to the controls<br>- Correlation with AHI, anthropometric parameters, oxygen saturation, and sleep architecture in the mild OSA group
	[144]		n=50 with OSA *(AHI≥20/h)*<br>n=20 control		- P-selectin did not differ between the two groups<br>- P-selectin did not differ between OSA groups with and without excessive daytime sleepiness
**PMPs**	[139]		n=12 with OSA *(AHI≥10/h)*<br>n=6 control	n/a	- PMPs levels did not differ between the two groups
	[148]	Case control	n=57 with OSA *(ODI>4%>7.5)*<br>n=15 control	n/a	- PMPs were higher in OSA group compared to the controls<br>- No correlation with ODI

	[149]	Case control / Interventional	n=27 with OSA *(AHI≥5/h)*<br>n=19 control	6 months of CPAP	- PMPs were higher in OSA group compared to the controls<br>- PMPs were higher in severe OSA group compared to the mild to moderate OSA group<br>- PMPs decreased after CPAP
	[166]	Randomized-controlled	n=23 with OSA (ODI>4%>10)<br>n=11 therapeutic CPAP n=12 subtherapeutic CPAP	2 weeks of CPAP withdrawal	- PMPs increased after CPAP withdrawal
	[167]	Randomized-controlled	n=41 with OSA (ODI>4%>10)<br>n=20 therapeutic CPAP<br>n=21 subtherapeutic CPAP	2 weeks of CPAP withdrawal	- No change after CPAP withdrawal
**Platelet count**	[150]	Case control	n=105 with OSA *(AHI≥5/h)*<br>n=41 control	n/a	- Platelet count did not differ between the AHI groups (control-mild-moderate-severe)<br>- No correlation with AHI
	[151]	Case control	n=87 with OSA *(AHI≥1/h)*<br>n=65 control<br>(only children)	n/a	- Platelet count was higher in OSA group compared to the controls<br>- Platelet count correlated with AHI, mean $SatO_2$, min $SatO_2$, ODI, AI, TST90% and sleep efficiency
	[152]	Case control	n=67 with SBD<br>n=45 with OSA<br>n=22 with snoring<br>n=70 control<br>(only children)	n/a	- Platelet count was higher in SBD group compared to the controls
	[168]	Interventional	n=61 with OSA *(AHI≥5/h)*	6 months after upper airway surgery	- Platelet count decreased after upper airway surgery
